# A python code for automatic construction of Fischer plots using proxy data

**DOI:** 10.1038/s41598-021-90017-9

**Published:** 2021-05-18

**Authors:** Daming Yang, Yongjian Huang, Zongyang Chen, Qinghua Huang, Yanguang Ren, Chengshan Wang

**Affiliations:** 1 State Key Laboratory of Biogeology and Environmental Geology, China University of Geosciences, Beijing, 100083 China; 2 School of the Earth Sciences and Resources, China University of Geosciences, Beijing, 100083 China; 3 Exploration and Development Research Institute of Daqing Oil Field Corporation, Daqing, 163712 Heilongjiang China

**Keywords:** Geology, Sedimentology

## Abstract

Fischer plots are widely used in paleoenvironmental research as graphic representations of sea- and lake-level changes through mapping linearly corrected variation of accumulative cycle thickness over cycle number or stratum depth. Some kinds of paleoenvironmental proxy data (especially subsurface data, such as natural gamma-ray logging data), which preserve continuous cyclic signals and have been largely collected, are potential materials for constructing Fischer Plots. However, it is laborious to count the cycles preserved in these proxy data manually and map Fischer plots with these cycles. In this paper, we introduce an original open-source Python code “PyFISCHERPLOT” for constructing Fischer Plots in batches utilizing paleoenvironmental proxy data series. The principle of constructing Fischer plots based on proxy data, the data processing and usage of the PyFISCHERPLOT code and the application cases of the code are presented. The code is compared with existing methods for constructing Fischer plots.

## Introduction

**Figure 1:**
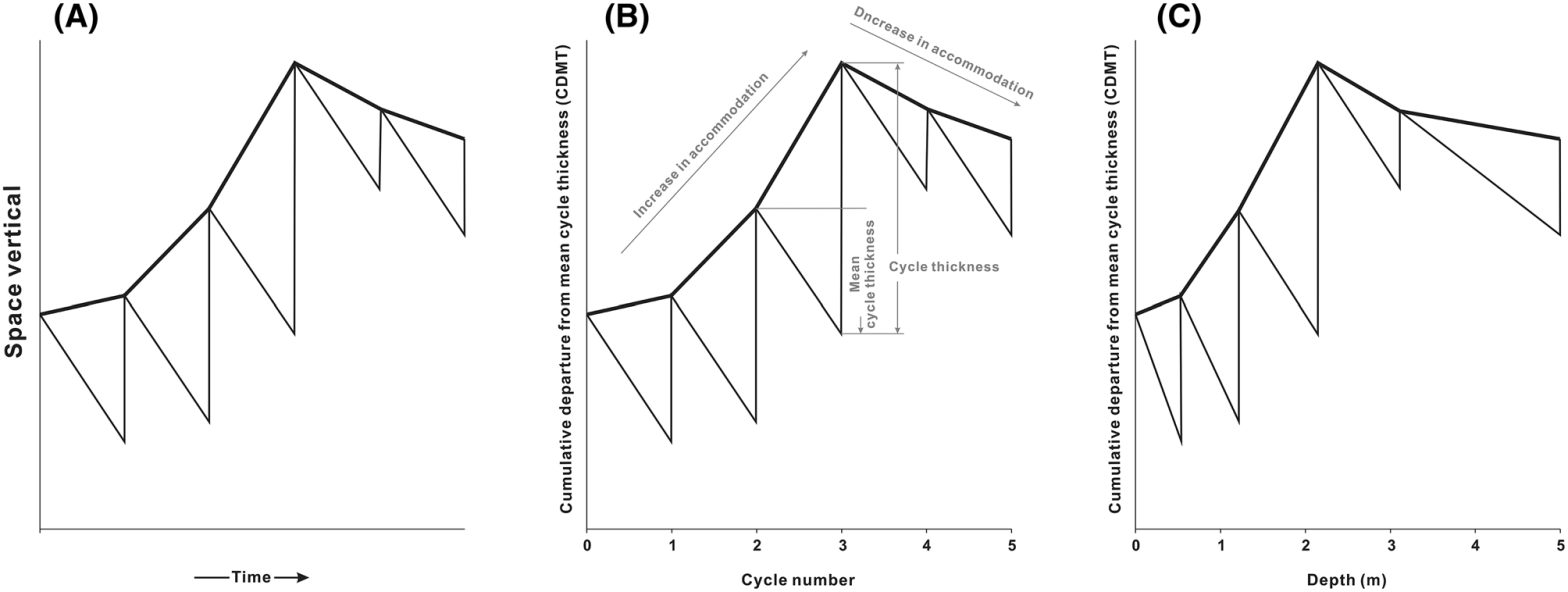
Original concept of Fischer plots introduced by Fischer (1964) (**A**). Schematic form of Fischer plot in time domain (**B**). Schematic form of Fischer plots in depth domain (**C**). Modified from Husinec et al., 2008^9^.

Sea-level change is an essential concept in paleoenvironmental research and petroleum exploration. Fischer plots are widely used to signify sea-level changes through representing cumulative cycle thickness after linear correction (i.e. minus average thickness) over cycle number or stratum depth (Fig. 1)^1^. Meanwhile, correlation of multiple Fischer plots could be used to distinguish the influence factors (eustatic or tectonic) of the detailed sea-level changes observed in Fischer plots^2–4^. In addition to sea-level changes, Fischer plots can also indicate lake-level changes in the case of continental basin^5–8^.

Fischer plots were commonly established by the sedimentary cycles observed in the field-section records, which may be fragmentary and discontinuous. Thenceforward, it is difficult to obtain a unified and objective result on the number and thickness of sedimentary cycles in the same strata with little lithological change. Some paleoenvironmental proxy data (e.g. natural gamma-ray logging data, elemental ratios, grain size, magnetic susceptibility) are potential materials to construct Fischer plots because cycles in these data series could be identified accurately by mathematical methods. Wire-line well logs and drilling core analysis, which have been collected in large quantities due to petroleum exploration, could provide abundant high-resolution and continuous subsurface data for constructing and correlating Fischer plots^1,3,6,7,10–13^.

Nonetheless, it is time-consuming and laborious to count the cycles preserved in geological data manually and map the Fischer plots with these cycles. To solve this problem, in this paper, we present an easy-to-use Python code “PyFISCHERPLOT” for constructing multiple Fischer plots based on the proxy data in batches. The cycles recorded in geological data from multiple sections or wells could be identified and the corresponding Fischer Plots based on these cycles could be graphed in time and depth domains through one-time operation of the code. We also introduce the principles of Fischer plots based on paleoenvironmental proxy data. The application cases of the code are presented through the study of correlation of Fischer plots in the lacustrine Nenjiang and Qingshankou Formations of the Songliao Basin, northeast China and the shallow-marine Sete Lagoas Formation of the São Francisco Basin, east-central Brazil. In addition, the validity of the code and the method is demonstrated by comparison with Fischer plot constructed by conventional methods.

## Theoretical background

### Sea-level change and Fischer plot

Relative sea level is defined as the height of geoid minus the solid rock or sediment surface height. Relative sea level changes are equal to the difference between the changes in sea surface height and solid surface height which create eurybatic and eustatic sea-level changes respectively^14–16^. Eurybatic sea-level changes in an ocean basin are always local, while eustatic sea-level changes are usually synchronous in regional, and could be theoretically correlated between ocean basins and even in global scale^14–16^. Relative sea-level oscillations generate changes of new accommodation spaces^17^, which could be filled by production of carbonate sediment, and consequently create variable thicknesses of sedimentary cycles in the shallow-marine carbonates. Therefore, after deducting a constant (minus average cycle thickness) to remove the solid rock subsidence, which is assumed changeless, the subsidence-corrected cumulative variable thickness of sedimentary cycles could represent eustatic sea-level changes^1,2,18,19^. So, new accommodation spaces being filled and a near-linear or constant subsidence are necessary assumptions for Fischer plots to define eustatic sea-level changes. However, the assumption for near-linear or constant subsidence usually cannot be determined, so Fischer plots are considered to indicate the changes of accommodation space (i.e., relative sea-level changes represented as the sum of subsidence and eustasy) in many studies^19^. Thus, we suggest that it is necessary to estimate whether a Fischer Plot indicates eustatic sea-level changes or relative sea-level changes based on its geological settings and data in the specific research case.

In the original concept of Fischer plots, the cumulative cycle thickness, which minus average thickness (vertical axis), was mapped onto a diagram in the time scale (horizontal axis) to illustrate sea-level changes (Fig. 1A)^2,20^. However, it is unlikely to ensure all sedimentary cycles in a stratigraphic record were formed over the absolute time interval, so that the horizontal axis of Fischer plots has been modified to cycle number (time domain) or stratum thickness (depth domain) (Fig. 1B,C). The vertical axis has been modified to a subsidence-corrected value labeled “Cumulative Departure From Average Cycle Thickness (CDMT)”^1,18^. The value of the CDMT at any cycle should be the difference between accumulation of each cycle thickness from the first cycle to the current one and average cycle thickness. During the period of sea-level rise, Fischer plots show a positive excursion in vertical axis and vice versa. The algebraic formula (5) for calculating CDMT was introduced^1^, and here we present the derivation process of this formula:

1 $$CDMT_{1} = D_{0} - D_{1} - \frac{{D_{0} - D_{N} }}{N},$$

2 $$CDMT_{2} = CDMT_{1} + (D_{1} - D_{2} ) - \frac{{D_{0} - D_{N} }}{N} = D_{0} - D_{2} - 2\left( {\frac{{D_{0} - D_{N} }}{N}} \right),$$

3 $$CDMT_{3} = CDMT_{2} + (D_{2} - D_{3} ) - \frac{{D_{0} - D_{N} }}{N} = D_{0} - D_{3} - 3\left( {\frac{{D_{0} - D_{N} }}{N}} \right),$$

4 $$CDMT_{i} = CDMT_{i - 1} + (D_{i - 1} - D_{i} ) - \frac{{D_{0} - D_{N} }}{N} = D_{0} - D_{i} - i\left( {\frac{{D_{0} - D_{N} }}{N}} \right),$$

5 $$CDMT_{i} = \left( {D_{0} - \frac{i}{N} \cdot T} \right) - D_{i} .$$

In this final form of the formula, *i* which stands for the current cycle number should start with *i* = 1 as the deepest complete cycle. *N*, *D*_i_, *D*_0_ and *D*_N_ stand for the total number of cycles, the actual depth of the *i*th cycle top, the depth of the base of the first cycle, and the actual depth of the last cycle top, respectively. *T* is the sum of thickness of the whole field section or well (equal to *D*_0_ − *D*_N_)^1^.

The Fischer plots were originally and widely used in studies on peritidal carbonate successions^1,2,18,19,21^, and then have been also used in variety of shallow-marine situations from shallow subtidal to deep subtidal facies and in wider geologic time-scale from Neoproterozoic to Cenozoic in some studies^13,22–31^. In several research cases, Fischer plots were used in lacustrine basins as indicators for lake-level changes^5–8^.

### Fischer plots in lacustrine environment

Relative lake-level changes are mainly controlled by the changes of lake surface height and tectonic subsidence^17^. After the tectonic subsidence are removed as a constant, Fischer plots could be used to define relative lake-level changes (represented as the sum of subsidence and lake surface height changes) in continental basins. Consistent with the marine environment, the application conditions of the Fischer plots in lacustrine environment are also the assumptions that the accommodation newly created was filled and the rate of subsidence was constant are satisfied.

According to the characteristics of lithofacies, lake basin could be divided into three filling types: overfilled lake basin, balanced-fill lake basin and underfilled lake basin^32^. In balanced-fill or overfilled lake basins, source recharge were sufficient to fill the new accommodation spaces, satisfying the necessary assumption for Fischer plots. In the absence of large-scale tectonic movement transforming the provenance of the basin, lake-level oscillations could generate synchronous cyclic changes in the distance between lake shoreline and sampling site, which in turn could synchronously create sedimentary cycles in grain size or sand-shale ratios at sampling site. Since the new accommodation space could be filled, observed sedimentary-cycle thickness in balanced-fill or overfilled basins subtracting subsidence could be interpreted to evaluate the accommodation space formed by relative lake-level changes. Thus, Fischer plots could be constructed by sedimentary cycles in balanced-fill or overfilled lake basins and interpreted to reflect relative lake-level changes.

### Fischer plots utilizing proxy data as material

Sedimentary cycles usually used to construct Fischer plots are derived from visual observation of field-section records. However, short-term, minor sea- or lake-level oscillations could also synchronously generate cyclic changes in detrital-inputs contents and paleowater conditions (such as redox and salinity). In terrigenous deposits, sand-mud ratios and grain sizes could be controlled by high-frequency sea- or lake-level cycles. And these cyclic changes formed by sea- or lake-level changes could be revealed by corresponding proxy data, such as natural gamma-ray logging and elemental ratios of drilling core, and used to construct Fischer plots.

**Figure 2:**
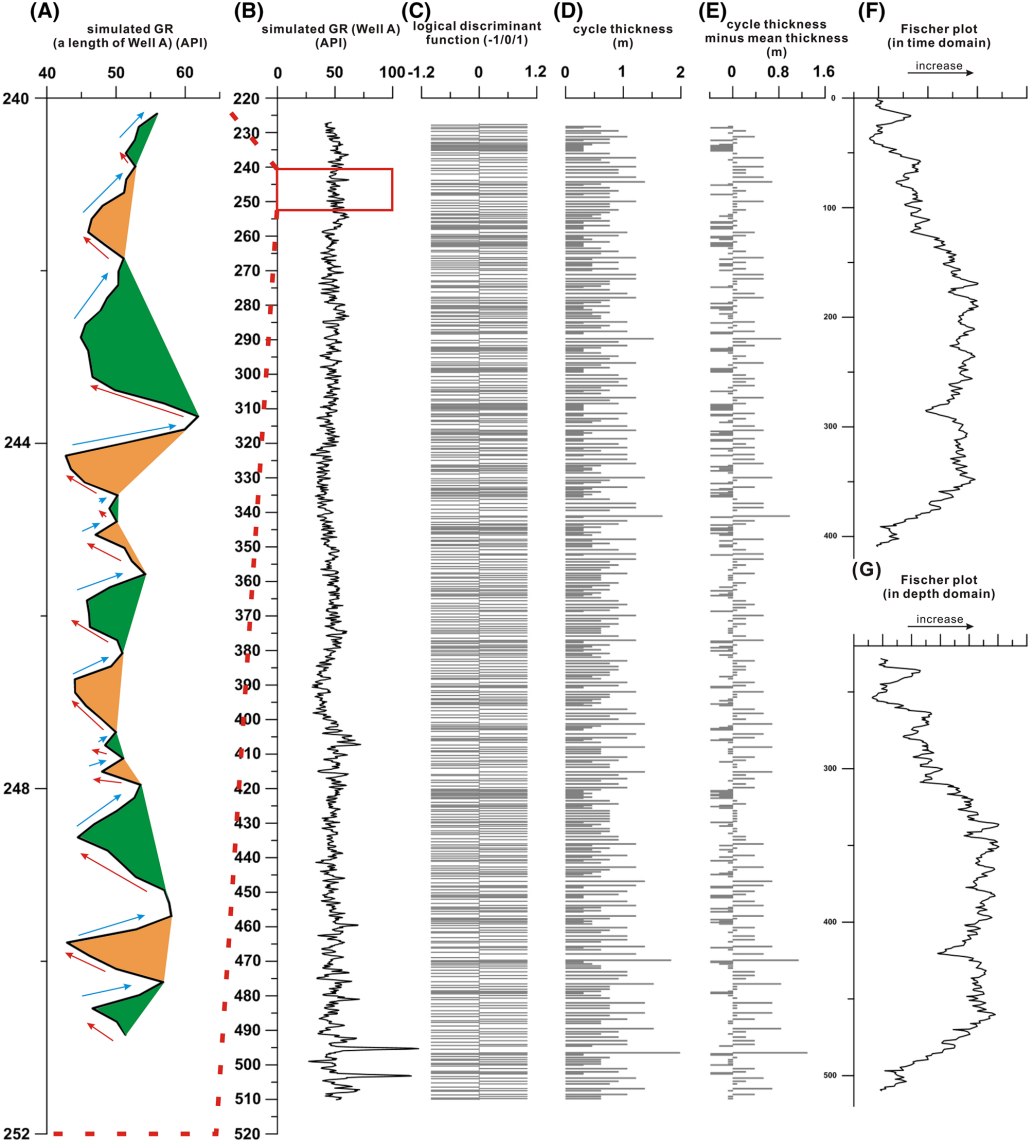
The process of constructing Fischer plots using natural gamma-ray (GR) data. Cycles in GR data are partly illustrated in alternating orange and green colors (**A**). Original GR data (test data) of the simulated Well A (**B**). The processes of calculating number and thickness of sedimentary cycles (**C**), (**D**,) and (**E**). Fischer plots of the Well A in both the time and depth domains (**F**) and (**G**).

In an continent basin, increasing trend of natural gamma-ray logging records could indicate higher clay contents and lower sand-shale ratios in sediments due to deeper relative lake level. When lake level becomes shallower, higher sand-shale ratios could be reflected by a decrease in natural gamma-ray values^11,33–35^. In shallow-marine carbonates, sea-level changes control gamma-ray logging changes in a similar way, as lower natural gamma-ray values are linked with carbonate-rich sediments. Thus, each sedimentary cycle in lacustrine or marine sedimentary records could be identified by an interval of a natural gamma-ray value rise followed by a decline. Cycles from 240 to 250 m in the simulated natural gamma-ray logging (Well A, Fig. 2B) are marked as an example (Fig. 2A). After the thickness of each cycle is calculated, Fischer plots based on these cycles could be constructed. The PyFISCHERPLOT code can implement this process. The equidistant (or nearly equidistant) sampling intervals should be used for these proxies, because non-equidistant data may affect the accuracy of cycle thickness.

In the case of marine or lacustrine terrigenous deposits, elemental ratios, such as Zr/Al, could indicate grain-size fluctuations caused by high-frequency sea- or lake-level cycles, since clays are rich in Al and silts in Zr^36^. Shallower sea or lake level could lead to larger grain size and higher values Zr/Al in sample site.

Apart from natural gamma-ray logs and elemental ratios, other proxies of shallow-marine and lacustrine deposits which controlled or influenced by sea- and lake-level changes (or environmental variations due to sea- and lake-level changes) could theoretically be used to construct Fischer Plots in same way.

### Correlation of multiple Fischer plots

Theoretically, Fischer plots are considered as a representation of subsidence-corrected (eustatic) sea-level changes. However, local vertical tectonic movements (controlled by solid-Earth factors) may affect the number and thickness of cycles in individual sampling sites, and then the pattern of corresponding Fischer plots^3^. From a single-well or -section study, it is unlikely to determine whether the changes in Fischer plots are subject to tectonic movements or eustatic sea-level changes. However, eustatic sea-level changes could be identified in all Fischer plots and correlated on regional or global scale through the isochronous boundaries (such as maximum flooding/regressive surface, system tract boundary and sequence boundary). Hence, the Fischer plot correlations based on these isochronous boundaries could be used to distinguish eustatic sea-level changes from local tectonic influences. In lacustrine conditions, lake-level (basin-scale lake surface height) changes could also be distinguished from tectonic influences through multiple-well or -section study with the same method (see Section Fischer plots of the Nenjiang Formation).

## Description of the PyFISCHERPLOT code

The PyFISCHERPLOT code (recorded in python file PyFISCHERPLOT.py) is written in Python 3.0 and it calculates the number and thickness of sedimentary cycles in multifarious geological proxies. It also provide numerical and graphical output for Fischer plots based on these cycles. The calculating and mapping processes and usage of the PyFISCHERPLOT are introduced in this section, using natural gamma-ray logs from Well A (test data in appendix), Well T11, Well S401 and Well L26, as an example.

### Data processing in the PyFISCHERPLOT code

The highest resolution individual cycles recorded in natural gamma-ray logs can be restricted by two adjacent spikes in natural gamma-ray logging values. The difference between depths of arbitrary two adjacent spikes in natural gamma-ray logging data is equal to the thickness of the corresponding sedimentary cycle^3^.

By running the PyFISCHERPLOT code, all the spikes in natural gamma-ray data could be identified using the methods of logistic discriminative function and first order difference (Fig. 2C). The differences between all the two adjacent spikes could be calculated in order by the method of first order difference and used as the thicknesses of cycles at smallest scale, thus the thicknesses of all sedimentary cycles are obtained (Fig. 2D). These sedimentary cycle thicknesses are accumulated in order from the bottom of the well to the top and then subtract mean thickness to obtain the series of “CDMT” (Fig. 2E). The resolution of the thicknesses of cycles identified from the proxy data depends on the resolution of these data. For example, the cycles identified from GR data of Well A show a mean thickness of ~ 1.2 m.

The cycle number is marked in order from the bottom to the top for all the cycles (the first cycle number is 1) as the horizontal axis of the Fischer plot in time domain (Fig. 2F). On the other hand, the depths of all cycles are counted as the horizontal axis of the Fischer plot in depth domain (Fig. 2G).

### Calls to the PyFISCHERPLOT code

The PyFISCHERPLOT code runs on Microsoft Windows operating system. Usage of the PyFISCHERPLOT is given below. To avoid confusion, the commands that have to be entered for this operation are indicated in *italics*.

**Figure 3:**
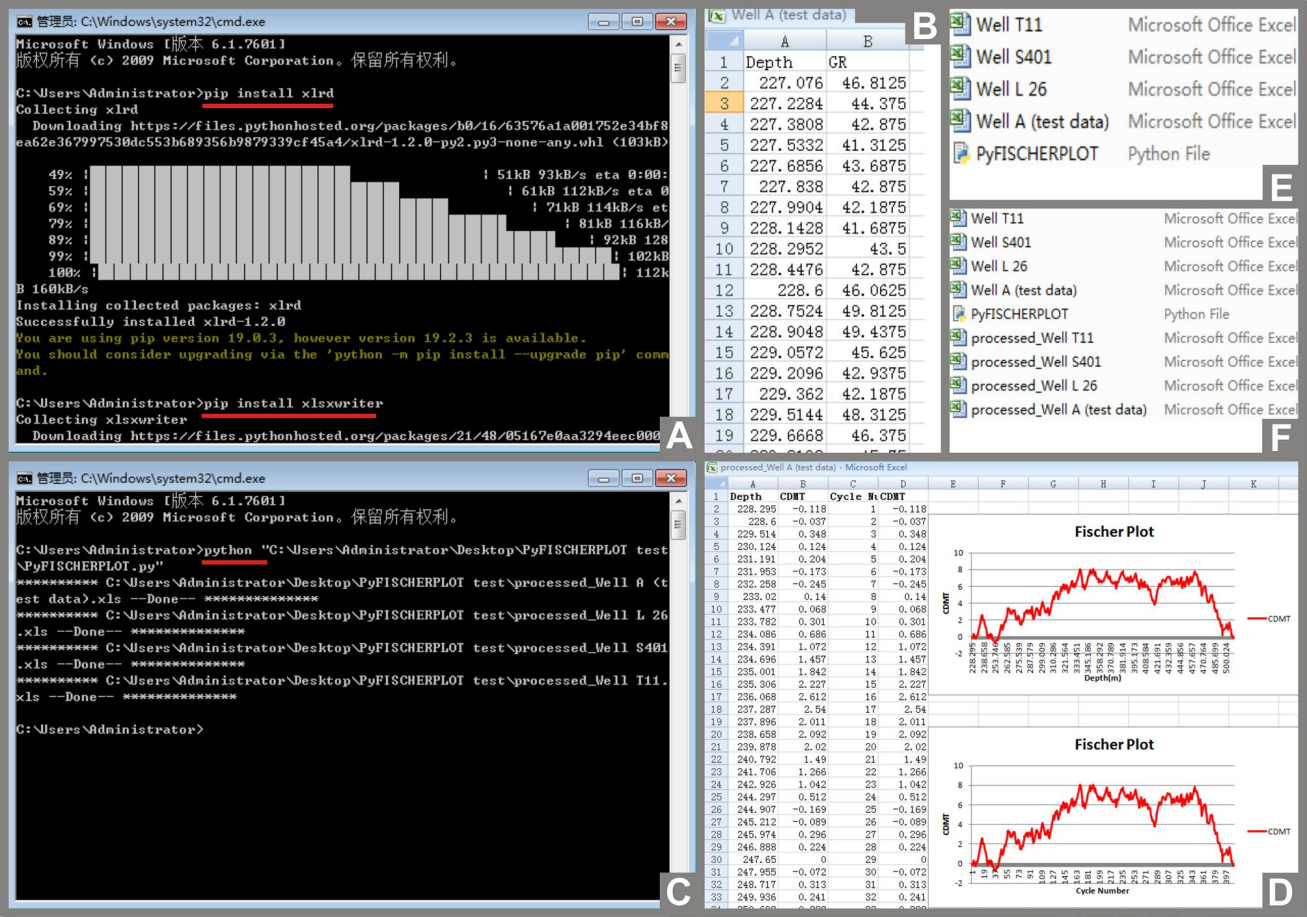
Demonstration of steps of using PyFISCHERPLOT.py file. Entering *pip install xlrd* and *pip install xlsxwriter* in the command prompt window to install Python libraries (**A**). Placing the PyFISCHERPLOT.py file and the Excel files which contain natural gamma-ray data of each well (Well A, Well T11, Well S401, Well L26) in the same path (folder) (**B**), (**E**). Typing in *python* and pressing the Space key, then dragging the PyFISCHERPLOT.py file onto command prompt window to run the PyFISCHERPLOT.py file (**C**). New Excel files containing the Fisher plot of each well in both the time (i.e. cycle number) and depth domains are established by PyFISCHERPLOT code in the same path (**D**), (**F**).

Before using the code, Python 3.0 language pack and two Python libraries (Xlrd and Xlsxwriter) have to be installed. In the process of installing python 3.0, the option "Add Python to PATH" needs to be checked. Python libraries Xlrd and Xlsxwriter are used for reading, writing Excel files and inserting graphs into Excel files, respectively. After Python 3.0 is installed, users need to press the Winkey and R keys together, and enter *CMD* to open the command prompt window, and then type into *pip install xlrd* and *pip install xlsxwriter* in the command prompt window and press Enter key respectively to install Python libraries Xlrd and Xlsxwriter (marked with red lines in Fig. 3A). After Python 3.0 and the two python libraries are installed, users could run the PyFISCHERPLOT code whenever necessary.

The input data files are Excel files with original data of each well. The values of natural gamma-ray content of each well and sampling depth (ascending series) have to be included in one-to-one correspondence (without null values) in two columns in a separate Excel file (XLS format), and all these Excel files to be processed (Well A, Well T11, Well S401, Well L26) have to be placed in the same path (folder) with the PyFISCHERPLOT.py file (Fig. 3B,E). Note that, the code does not directly work with Log ASCII Standard (LAS) files. In the case of field section sampling, sampling height should be converted to depth below top of the section. Then double-clicking the Pyfischerplot.py file to let the code start to calculate. Once the calculation is complete, new Excel files containing the Fisher plots in both the time (i.e. cycle number) and depth domains of each well are created automatically as the output data files (Fig. 3D,F). The corresponding values displayed by the Fischer plots on the horizontal and vertical axes are also saved in these output Excel files (Fig. 3D). If double-clicking can not let the code start calculating due to the individual system settings, users should open a command prompt window, and type in *python* and press space key, then drag the PyFISCHERPLOT.py file into the command prompt window (marked with a red line in Fig. 3C) to run the code.

The PyFISCHERPLOT code can process data from any number of wells with one-time operation. The code, test data file, out put file and demo video of usage could be downloaded from the link of appendix materials at the end of this paper.

## Application cases

Fischer plots of Members 1 and 2 of the lacustrine Nenjiang Formation of the Songliao Basin, northeast China are established based on 18 natural gamma-ray logs and core-scanning Zr/Al ratios of the International Continental Drilling Program SK1. The Zr and Al ratios of the SK1 core were evaluated at the China University of Geosciences using an Itrax X-ray Fluorescence Core Scanner and CIT-3000SMQ spectrometer. Fischer plots of the shallow-marine Sete Lagoas Formation in the São Francisco Basin are established based on elemental geochemical data. All the natural gamma-ray logging data (gray curves) and geochemical data (gray scatters, jade-green curves) are processed by the PyFISCHERPLOT code in a single operation to generate Fischer plots (black curves, green curves), and then modified by CorelDRAW X4 (Figs. 5, 6).

### Fischer plots of the Nenjiang Formation

**Figure 4:**
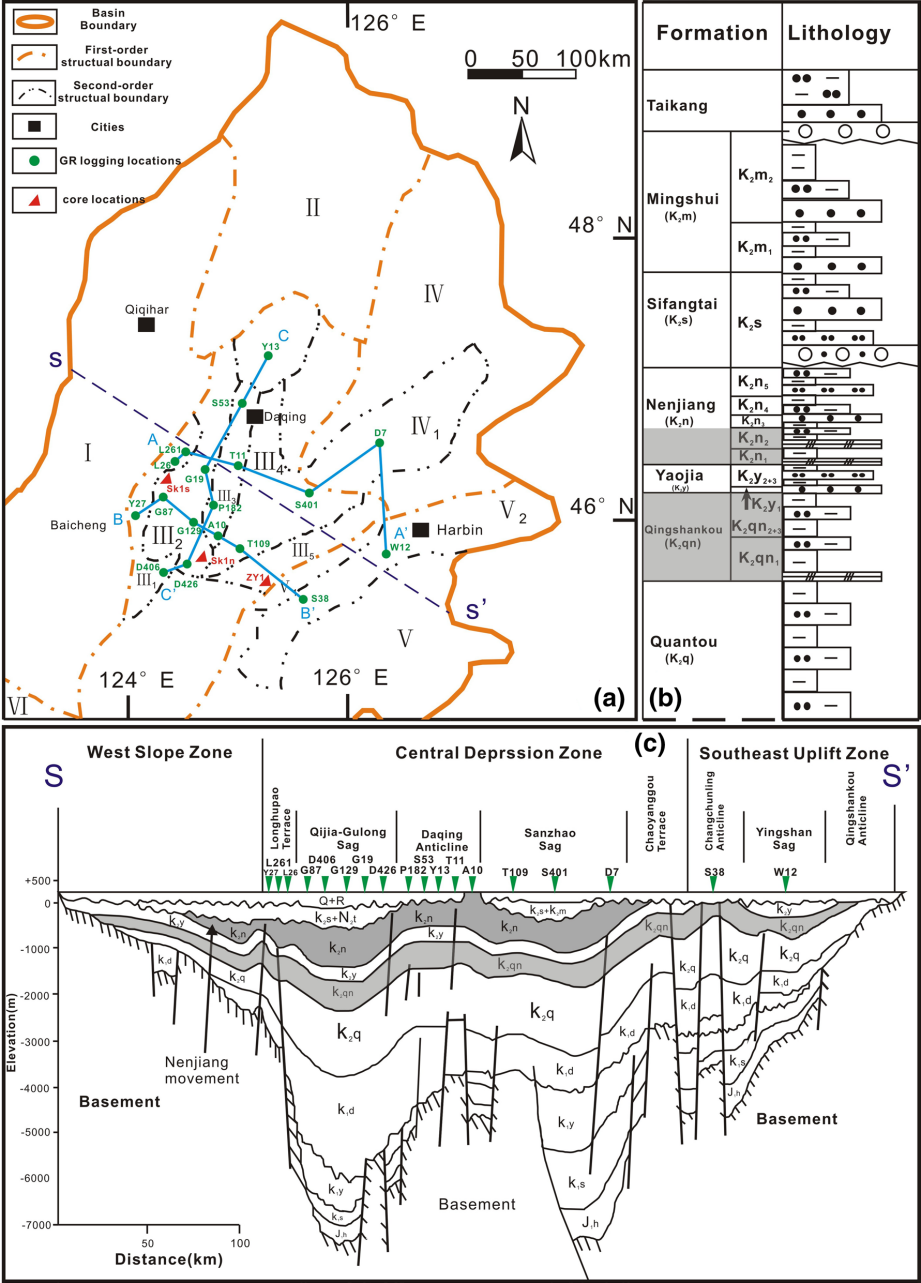
Map of structural units of the Songliao Basin with locations of natural gamma-ray logs (**a**). I, western slope zone; II, northern plunge zone; III, central depression zone; III1, Longhupao-Honggang Terrace; III2, Qijia-Gulong Sag; III3, Daqing Anticline; III4Sanzhao Sag; III5, Heiyupao Sag; IV, northeastern uplift zone; IV3, Suihua Sag; V, southeastern uplift zone; V1, Binxian-Wangfu Sag; VI, southwestern uplift zone. Lithostratigraphy of the Upper Cretaceous Songliao Basin (**b**). Stratigraphic cross section (S–S′) through the basin (**c**). Modified from Yang et al., 2018^7^.

The Songliao Basin located in northeast China is an intra-cratonic sedimentary rift basin filled by Cretaceous lacustrine deposits. Members 1 and 2 of the Nenjiang Formation, which contain the main source rock intervals of the basin, mainly consist of black, gray-green mudstones and shales (Fig. 4b)^7,37,38^. The basin was a balanced-filled basin during the deposition of the basal Nenjiang Formation^39^, and had brief depositional periods (~ 2.3 Ma)^40^ and stable tectonic environment^41^. Hence, we estimate that its subsidence rate was nearly constant or linear at that time and Fischer plots are reasonable for indicating “eustatic” lake-level changes (lake surface height changes). Tectonically, the basin can be divided into 6 first-order tectonic units (the central depression zone, north plunge zone, west slope zone, northeast uplift zone, southeast uplift zone, and southwest uplift zone) and 31 s-order tectonic units (Fig. 4c)^41^. Eighteen natural gamma-ray logs in 8 secondary tectonic units of the central depression zone, northeastern uplift zone and southeastern uplift zone are collected for constructing Fischer plots (Fig. 4).

**Figure 5:**
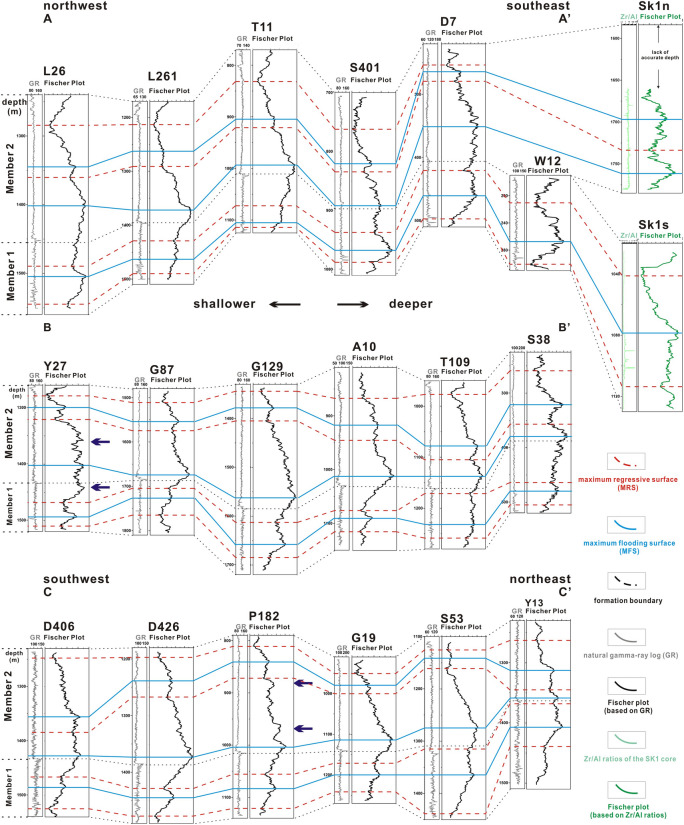
Three cross-sections (**A**–**A**′, **B**–**B**′ and **C**–**C**′) of Fischer plots based on natural gamma-ray logs. Sampling positions are shown in Fig. 4. Blue lines and red dashed lines represent correlatable maximum flooding/regressive surfaces. Blue arrows indicate local tectonics controlled influences.

Three cross-sections of these Fischer plots based on natural gamma-ray logging are established through maximum flooding/regressive surfaces (MFS/MRS) (Fig. 5). A total of three maximum flooding surfaces and four maximum regressive surfaces have been identified from correlations of Fischer plots. Curves of Fischer plots based on Zr/Al ratios of the SK1 have same variations and could be correlated to the natural gamma-ray logging Fischer plots. That is to say three complete transgression-regression oscillations occurred in the Members 1 and 2 of the Nenjiang Formation. On the whole, the lake level was comparatively deeper at the bottom of Members 1 and 2, corresponding to the oil shale intervals of the Members 1 and 2, which are considered to be formed in deeper water environment^37^. Because changes in subsidence rate could be excluded based on its brief depositional periods, it is possible that variations in precipitation contributed to the varying lake levels in different periods. In addition, the marine transgression that occurred in the basal Nenjiang Formation may also be the cause of lake-level changes and the formation of oil shale^37^. In the well sites, where single or partial Fischer plots show inflection points that could not be isochronously correlated in all the Fischer plots, local tectonic events may have occurred and lead to these local differences between the wells (marked with blue arrows).

Some researchers consider Fischer plot as a quantitative method to indicate sea-level (or lake-level) changes^2^, but the results of our data contradict this view. Most Fischer plots indicate that the lake level was the deepest at the first MFS in Member 2 of the Nenjiang Formation, followed by the MFS in Member 1, and the shallowest at the second MFS in Member 2 where no oil shale was formed. However, Fischer plots of Wells S401, Y13 show a deepest lake level in Member 1, and Wells A10, G87, D426, D426, S38 and T109 show shallowest lake level in Member 1. The different local hydrology or landform of individual wells could result in these differences. In addition, tectonic movements could also change the natural pattern of sedimentary cycles in local position, and then affect the pattern of Fischer plot^3^. Thus, the tectonic movements occurred during the structural inversion period beginning at the end of the Nenjiang Formation may changed the original sedimentary records of basal Nenjiang Formation, and then caused these detailed differences in individual Fischer plots. We could only use the common features of the majority of the Fischer plots to estimate the relative heights between the MFSs identified by the three cross-sections. Since the general trend of lake-level changes could be identified, whereas the relative heights between the MFSs cannot, we suggest that it is more accurate to regard Fischer plot as a semi-quantitative method indicating sea- or lake-level changes.

### Fischer plots of the Sete Lagoas Formation

**Figure 6:**
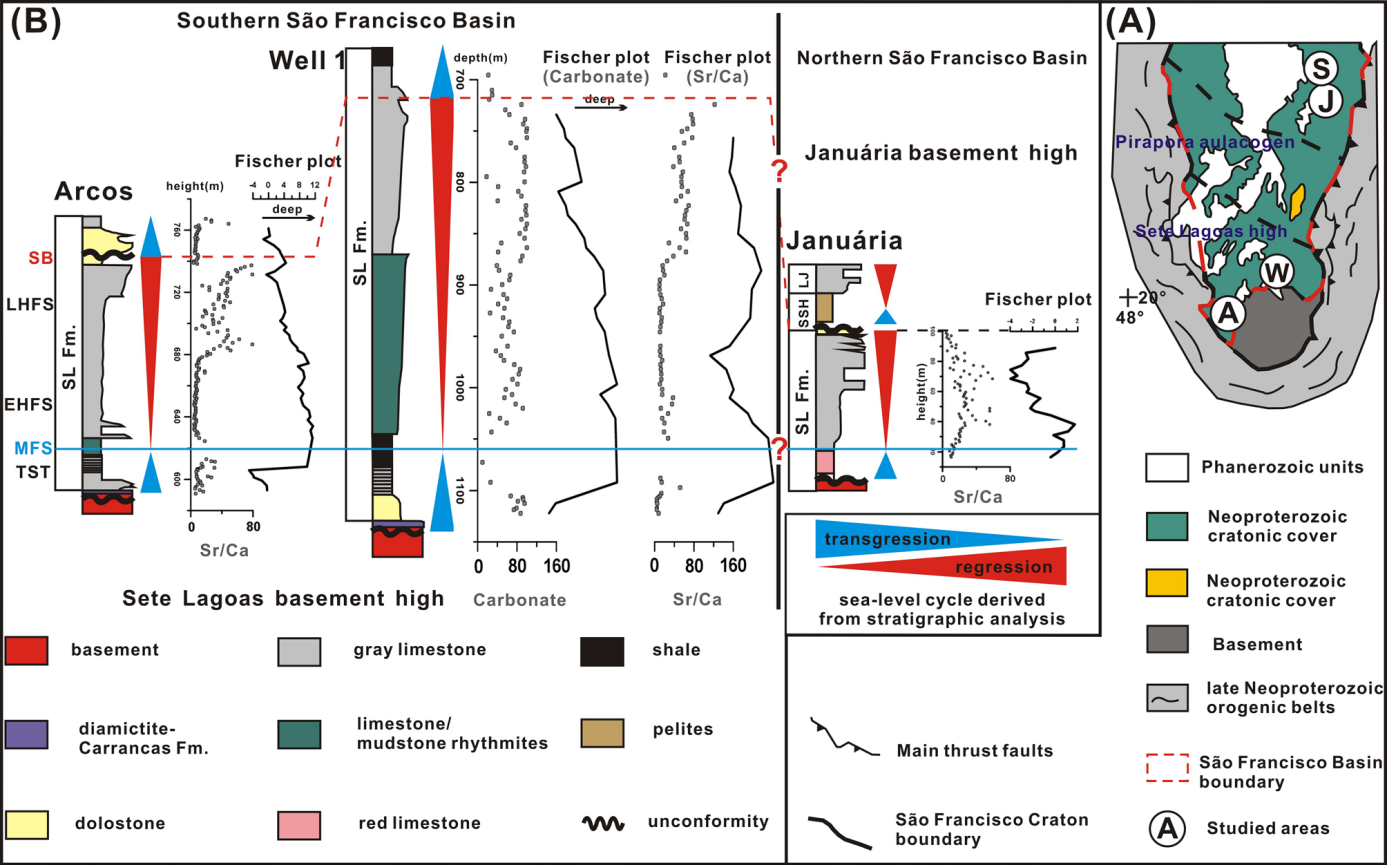
Geological map of the São Francisco Basin and locations of sampling sections (**A**) (Ref.^42^). Fischer plots based on Sr/Ca ratios and carbonate content vs. sea-level cycle derived from stratigraphic analysis. *MFS* maximum flooding surface, *SB* sequence boundary, *TST* transgressive system tract, *EHST* early highstand system tract, *LHST* late highstand system tract.

The polycyclic intracratonic São Francisco Basin located in the east-central Brazil records tectonosedimentary evolution from the Paleoproterozoic to the Mesozoic. The carbonate-dominated Sete Lagoas Formation (basal Bambuí Group) have been interpreted as a shallow-marine carbonate ramp^42^. The sequence stratigraphy and Sr (Sr/Ca), carbonate contents of the Sete Lagoas Formation are investigated from the Arcos section and Well 1 in the Sete Lagoas Basement High, south of the basin, and the Januaria section and Santa Maria da Vitoria section in the Januaria Basement High, north of the basin (Fig. 6)^42^.

A number of researches have reported the validity of Fisher plots for deriving relative sea-level changes in carbonate ramp environment^4,13,23,28,30,31^, but never in the Bambui Group in Brazil. Sea-level changes can cause large variations in strontium contents and Sr/Ca ratios of seawater, and thus in marine carbonates^43–45^. Thus, four Fischer plots based on Sr/Ca ratios and carbonate content from Januária section, Arcos well and Well 1 are constructed to illustrate relative sea-level changes of each section or wells (Fig. 6). Data set of the Santa Maria da Vitória section was discarded because of its changeful sample interval. Before running the PyFISCHERPLOT code, the height of sampling points (descending series) of the Januária and Arcos sections in original data sets was converted to the depth below the top of the section (ascending series) for calculation, and then we converted the vertical axis of its Fischer plot back to height in Fig. 6 for correlation with other sections.

The three carbonate successions of the lower-to-upper Sete Lagoas Formation all present a shallowing of depositional conditions according to integrated stratigraphic-chemostratigraphic analysis^42^, and evolve from maximum flooding surface to early highstand system tract and then to late highstand system (Fig. 6). And transgressive pattern briefly occur at the base and top of the Sete Lagoas Formation. After brief increases at the basal Sete Lagoas Formation (transgression systems tract), sea levels of the three successions indicated by the four Fischer plots all reached a maximum depth at the lower Sete Lagoas Formation, which corresponds to the maximum flooding surface indicated by respective stratigraphic analysis. Above the maximum flooding surface, the three Fischer plots of Arcos well and Well 1 represent gradual decreases in relative sea level, which is consistent with the conclusion of regression derived from stratigraphic analysis. Although the Fischer plot of the Januária section indicate a brief sea-level rise at the bottom of the Sete Lagoas Formation, the overall sea-level changes indicated by the Fischer plot still show a shallowing-upward trend. Therefore, we suggest that the sea-level changes indicated by the Fischer plots of the Sete Lagoas Formation are consistent with evolution of the sequence and systems tracts indicated by respective stratigraphic analysis of the three carbonate successions^42^.

The Arcos section and Well 1 in the Sete Lagoas Basement High and the Januaria section and the Januaria Basement High were correlated through the SBs and MFSs indicated in respective sites by stratigraphic analysis (Fig. 6). Although the Fischer plots of each section or well support the presence of MFS and SB for that site, the correlation between Sete Lagoas Formations in the Sete Lagoas Basement High and Januaria Basement High is still uncertain (marked with ? in Fig. 6), because geochronology of the Sete Lagoas Formation is still in debate and There is currently no evidence that the Sete Lagoas Formations in Sete Lagoas Basement High and Januaria Basement High were formed in the same age^46–48^.

## Comparison to Fischer plots constructed by conventional method

**Figure 7:**
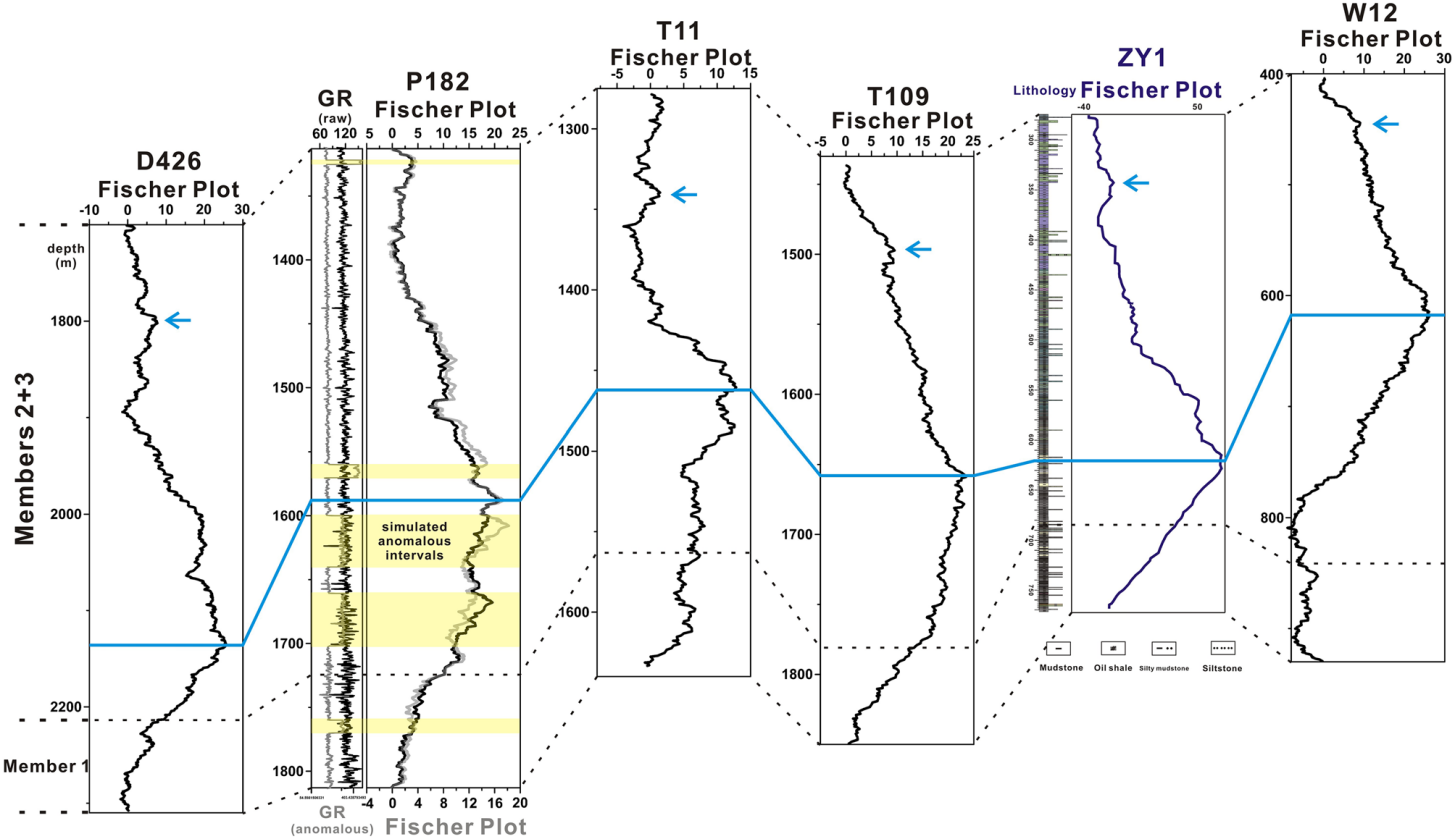
Comparison of Fischer plots constructed by conventional method and the PyFISCHERPLOT code. Sampling positions are shown in Fig. 4. The Fischer plot of Well ZY1 (purple curves) was constructed by cycles manually picked from the core^50^. The other Fischer plots (black curves) are constructed using GR logs by PyFISCHERPLOT. The simulated anomalous GR signal and corresponding Fischer plot of Well P182 are painted in gray and the yellow intervals stand for the abnormal values.

Previous studies proved that different cycle-splitting strategies applied to same stratigraphic interval will not affect the general CDMT pattern of the corresponding Fischer plot^18,49^. Even the gross form of the longer-period CDMT curves in Fischer plots constructed directly from individual lithologic units (individual beds) is not much different from those constructed from sedimentary cycles^49^. This is because, when cycles identified by one stratigrapher are split into thinner cycles by another, this cycle-splitting act generates clusters or consecutive runs of the thinner cycles rather than place them randomly into the section^18^. Compared with the conventional method, high-resolution proxy data, such as GR logging, could be used to delimit higher resolution coarsening (shallowing) and fining (deepening) cycles with higher resolution. Using PyFISCHERPLOT code for proxy data is similar to the thinner cycle-splitting strategy, and therefore should not affect the general accuracy of a Fischer plot. To test this, we compare Fischer plots based on GR logging to a conventional Fischer plot in the Songliao Basin (Fig. 7).

The Qingshankou Formation of the Songliao Basin mainly consists of dark mudstones and shales (Fig. 4b)^37,38^. The basin was a balanced-filled basin during the deposition of the Qingshankou Formation, which satisfies the necessary assumption of using Fischer plots^39^. The Fischer plot of the Qingshankou Formation was constructed by sedimentary cycles identified from the core of the Well ZY1 by conventional method (purple curves in Fig. 7)^50^. The Well ZY1 is located in the central depressed zone of the Songliao Basin (Fig. 4c)^50^. We construct Fischer plots of the same stratigraphic interval by GR logs of D426, P182, T11, T109 and W12 (black curves in Fig. 7). The CDMT curves of Fischer plots of the ZY1 and the five GR logs show the coincident pattern. Lake level rose in the Member 1 of the Qingshankou Formation and kept falling till the top boundary of the formation after reaching the maximum depth at bottom of the Members 2 + 3, which is indicated by a gradual decrease in CDMT values after an initial increase. In the lower part of Members 2 + 3, a slight bulge also appeared on the CDMT curves (marked by blue arrows in Fig. 7), which may indicate an inconspicuous lake transgression. Thus, we suggest the correlation of the Fischer plots further confirms the validity of constructing Fischer plots by higher resolution proxy data.

In addition, we randomly disrupted and amplified part of the GR data from Well P182 to simulate the anomalous GR signal due to the anomalous changes in stratigraphy, such as radioactive minerals (yellow intervals in Fig. 7). Comparing the Fischer plots based on simulated data (gray curves) and raw GR data of Well P182 (black curves), we found that the anomalous intervals have no obvious influence on the gross form of the Fischer plot. Nevertheless, the detailed form of the Fischer plot in these yellow intervals, has some differences, due to the presence of anomalous data. For example, the maximum values of CDMT, which occurred above the depth of 1660 m in the correct Fischer plot, are shifted to the anomaly interval below 1660 m in the simulated Fischer plot. We, therefore, suggest that if detailed correlations of strata with more abnormal intervals are needed, the abnormal intervals should be excluded when using PyFISCHERPLOT, or tools, such as Fischerlab software^51,52^, should be used to pick the cycles manually from materials.

## Comparison to other software

The first software for constructing Fischer plots was written in VS FORTRAN version 2.0 and need to need to input many parameters, such as, horizontal scale and vertical scale, total duration of plot, subsidence rate, average cycle period^53^. The Excel spreadsheet FISCHERPLOT simplifies the input data into cycle thickness and number to construct Fischer plot in both the time and depth domains^9^. The Matlab-based software Fischerlab is used to pick cycles manually from single wireline well-logs or stratigraphic data to construct Fischer plots in both the time and depth domains^51,52^. Owing to the Matlab platform, the FischerLab software accepts a wide range of input data formats including Log ASCII Standard (LAS), CSV and XSLX formats.

Unlike the above-mentioned software, which need to manually split the cycles of a single well or section, the Python code PyFISCHERPLOT could automatically identify the cycles in the data and output Fischer plots together with corresponding CDMT values in both the depth and time domains. This process can simultaneously deal with data from multiple wells or sections at the same time. Furthermore, in this study, we extend the range of input data from wireline logs and lithologic cycles to proxy data controlled by sea-level or lake-level changes. In the case of the Qingshankou Formation, the validity of PyFISCHERPLOT has been demonstrated. However, we suggest that manually cycle-splitting software, such as the Fischerlab, should be used in stratigraphic intervals with a large number of outliers or perturbations unrelated to base-level changes to obtain the detail form of the Fischer plots.

## Conclusion

Fischer plots as graphic representations of sea-level and lake-level changes are conventionally constructed from lithologic cycles. In this study, we provide the Python code PyFISCHERPLOT which automatically identifies the cycles in the input data using a discriminative function and the first-order difference method. This approach overcomes the time and labor-intensive disadvantages of the conventional method. In addition, the range of materials for constructing Fischer plots is extended from wireline logging and stratigraphic data to proxy data controlled by sea-level and lake-level changes. The code is open-source and available for download and use. The principle, usage and application cases of the code are introduced in this paper. The validity of the code and the method is demonstrated under the comparison to the conventional method in previous study and simulated abnormal input data. Due to the need for petroleum exploration and paleoclimatic research, data of cores and well logs have been collected in large quantities all over the world. We hope the PyFISCHERPLOT code together with other software, such as the Fischerlab, can use these data to make sea-level and lake-level reconstructions and correlations more efficient, and thus improve paleoclimatic research and oil exploration.

## Data Availability

All the appendixes (PyFISCHERPLOT code, test data file, out put file and demo video of usage) can be found at https://github.com/DamingYANG1991/PyFISCHERPLOT.git.
